# Lateral decubitus position to facilitate pelvic examination of the patient with severe obesity

**DOI:** 10.1186/s12905-021-01289-2

**Published:** 2021-04-07

**Authors:** Daniel M. Breitkopf

**Affiliations:** Department of Obstetrics and Gynecology, Mayo Clinic College of Medicine, 200 First St SW, Rochester, MN 55902 USA

**Keywords:** Uterine cervix, Pelvic anatomy, Patient positioning

## Abstract

**Background:**

Patients with elevated BMI pose a number of challenges for the gynecologist. Pelvic examination may be more difficult due to adiposity in the perineum and labia, increasing the distance between the vulva and cervix. The objective of the current work was to describe use of the lateral decubitus position to improve visualization of the cervix in women with severe obesity.

**Methods:**

A case series was collected. From 7/1/2010 until 1/31/2020, all records of patients with obesity and unsuccessful cervical visualization during pelvic exam in the dorsal lithotomy position in the author’s clinical practice were reviewed after obtaining Mayo Clinic Institutional Review Board approval. For the lateral decubitus position, the patient was asked to lie on her side on the exam table, facing away from the examiner with knees bent. An assistant elevated the upper bent leg 45 degrees from horizontal, exposing the perineum. A vaginal speculum was then placed in the vagina with the posterior blade toward the anus. The speculum was opened gently as would be done with examination in dorsal lithotomy position until the cervix was visualized.

**Results:**

Eleven patients with severe obesity in the gynecologic practice of the author with prior unsuccessful cervical visualization in dorsal lithotomy position were examined in the lateral decubitus position. In all but one case the cervix was successfully visualized in the lateral decubitus position and all intended intrauterine procedures were successfully performed.

**Conclusions:**

In this case series, the use of the lateral decubitus position appears to improve visualization of the cervix in the outpatient setting among women with severe obesity. Consideration should be given to use of the lateral decubitus position when the cervix cannot be visualized in the dorsal lithotomy position.

## Introduction

Obesity has become epidemic in the United States. From 2007 to 2016, the obesity rate in women increased from 35.4 to 41.1%, while the rate of severe obesity (BMI ≥ 40) increased to 9.7% [1]. Patients with elevated BMI pose a number of challenges for the gynecologist. Pelvic examination may be more difficult due to adiposity in the perineum and labia, increasing the distance between the vulva and cervix. Furthermore, the abdominal pannus limits the ability to palpate the uterus and ovaries. The abdominal pannus may also increase the vaginal pressure making speculum examination more difficult. Intra-abdominal pressure is markedly increased in patients with morbidly obesity. Intra-abdominal pressure is as much as 12 cmH_2_0 higher in patients with morbid obesity compared to patients of normal weight [2]. In general, intra-abdominal pressure and vaginal pressure are closely correlated.

Traditionally pelvic examination has been performed in the dorsal lithotomy position to facilitate access to the perineum and to adduct the thighs for bimanual examination [3]. Other positions for examination have been described including knee chest position, lateral decubitus (Sims) position, M position, V position and diamond position [4]. Little information is available on the comparative efficacy and patient's satisfaction with the various positions. The Sims position was originally described by J. Marion Sims in the 1800s for surgical procedures, and reportedly provides better visualization of the cervix than the dorsal lithotomy position [5].

As more minimally invasive procedures move to the office setting, development of techniques to facilitate ambulatory surgical practice are needed. The objective of this case series was to describe use of the lateral decubitus position to improve visualization of the cervix in women with severe obesity.

## Methods

All patients with obesity in the gynecologic practice of the author with prior unsuccessful cervical visualization in dorsal lithotomy position were examined in the lateral decubitus position. Unsuccessful cervical visualization was defined as the inability to see the cervical os despite use of a vaginal speculum in dorsal lithotomy positon. From 7/1/2010 until 1/31/2020, all records of patients meeting these criteria in the author’s clinical practice were reviewed after obtaining Mayo Clinic Institutional Review Board approval. All exams and testing were performed during one clinic visit.

**Fig. 1:**
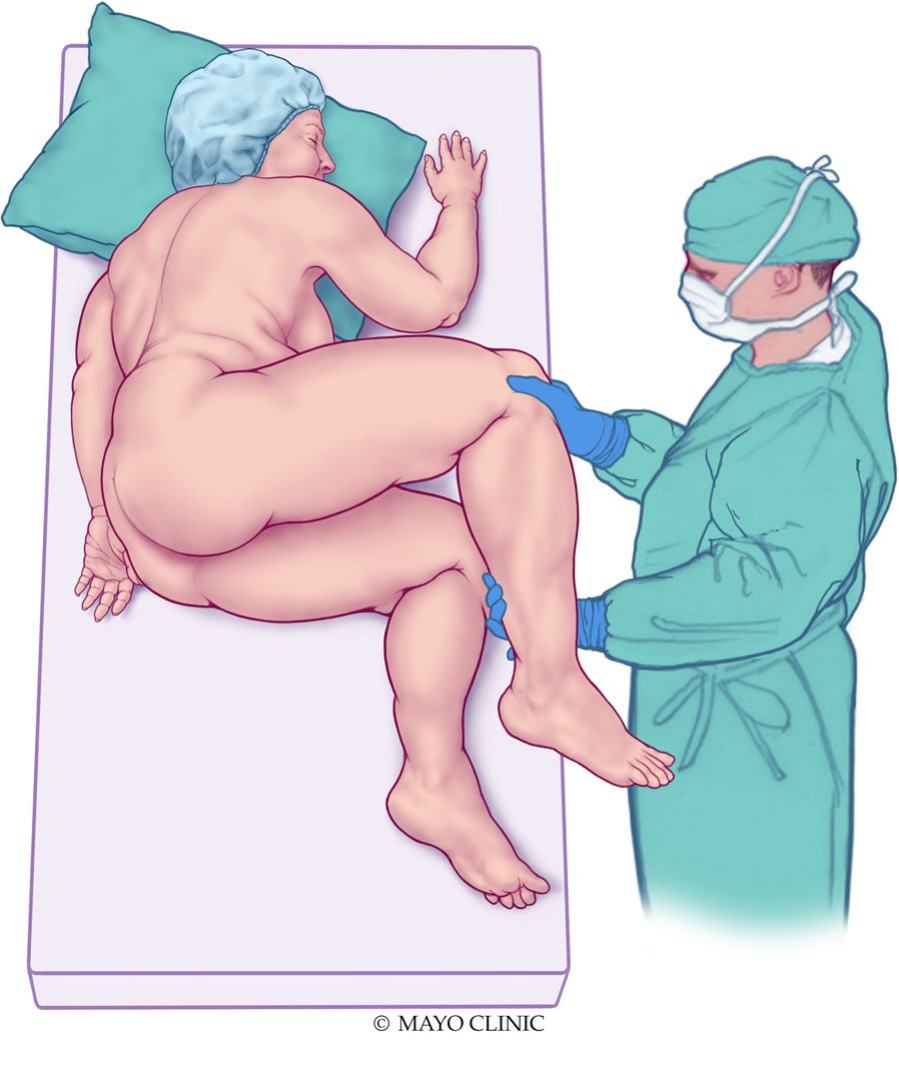
Lateral decubitus position for pelvic examination

As most patients had not been examined in positions other than dorsal lithotomy, the examiner explained the reasoning for exam in lateral decubitus position as well the details of how the exam is conducted. Sensitivity to patient concerns about embarrassment or body image were acknowledged during pre-procedure counseling. The patient was asked to lie on her side on the exam table, facing away from the examiner with knees bent (see Fig. 1). An assistant elevated the upper bent leg 45 degrees from horizontal, exposing the perineum. A large Weisman Graves reusable (metallic) vaginal speculum was then placed in the vagina with the posterior blade toward the anus. The speculum was opened gently as would be done with examination in dorsal lithotomy position until the cervix was visualized.

## Results

Twelve patients met inclusion criteria; however one was excluded due to lack of Minnesota research authorization. Minnesota law requires written authorization from patients prior to use of their medical records for research purposes. None of the patients had gynecologic conditions or history which would affect performance of a pelvic exam such as vulvar vestibulitis, vaginismus or dyspareunia. Results of use of the examination technique are presented in the Table 1.

**Table 1:** Patients with severe obesity examined in lateral decubitus position

Subject Number	Race/ Ethnicity	BMI	Para	Menopausal	Cesarean history	Indication	Cervix visualized	Procedures performed	Sample(s) adequate
1	White	58.1	3	Yes	No	Endometrial hyperplasia	Yes	Endometrial biopsy	Yes
2	White	48.5	1	Yes	Yes	PMB	Yes	Office hysteroscopy Endometrial biopsy	Yes
3	White	49.0	5	Yes	No	PMB	Yes	Office hysteroscopy Endometrial biopsy	No
4	White	60.1	2	Yes	Yes	Vaginal discharge	Yes	Pap test	Yes
5	White	51.8	0	Yes	No	PMB	Yes	Pap test Office hysteroscopy Endometrial biopsy	Yes
6	White	59.7	0	No	No	Irregular menstrual bleeding	Yes	Office hysteroscopy Endometrial biopsy	Yes
7	Latino	57.7	3	No	Yes	Heavy menstrual bleeding	Yes	Endometrial biopsy	Yes
8	White	62.1	3	Yes	No	PMB	Yes	Endometrial biopsy	Yes
9	White	60.8	2	Yes	Yes	PMB	No	Office hysteroscopy Endometrial biopsy	Yes
10	White	63.3	3	Yes	No	Endometrial hyperplasia	Yes	IUD insertion	N/A
11	White	63.2	0	Yes	No	PMB	Yes	Office hysteroscopy Endometrial biopsy	Yes

*PMB* postmenopausal bleeding, *N/A* not applicable

The average age was 59 years (range 41–75) and the average BMI was 57.7 (range 49–63.3). All but one patient had successful visualization of their cervix. Of the ten patients who had samples collected from the cervix or endometrium, the sample was adequate in all but one. In the case (subject #3) with an inadequate sample, office hysteroscopy revealed a normal uterine cavity with both tubal ostia visualized. Subject #9 did not tolerate lateral decubitus position due to discomfort. In this case, vaginoscopy was subsequently performed in the office and the cervix was successfully cannulated to visualize the uterine cavity.

## Discussion

The use of lateral decubitus position appeared to improve visualization of the cervix in women with morbid obesity. All patients had Class 3 (severe) obesity and the cervix was not able to be visualized by standard dorsal lithotomy examination technique. In most cases, the intended office procedure was performed successfully, avoiding the need for examination under anesthesia. Lateral decubitus position shifts the weight of the pannus, which may decrease intraabdominal and vaginal pressure. Similarly, the labial fat pad may shift away from the top blade of the speculum. These two factors may facilitate speculum examination in this population.

Examination in lateral decubitus position proved to be easy to adopt in office practice. Two assistants are needed; one to help hold the upper leg and the other to hand needed equipment to the examining clinician. There may be ergonomic risks to the health care provider holding the upper leg. Some of this risk might be mitigated by use of a standing stool for the assistant. Patient comfort was not formally assessed, but anecdotally, all but one patient in this case series tolerated the exam without significant difficulty. In cases where the cervix is unable to be visualized with a speculum, use of office based vaginoscopy may provide a viable alternative for some procedures [6]. Vaginoscopy is typically performed with a rigid or flexible hysteroscope, allowing visualization of the cervix and vagina, as well as the uterine cavity in many cases. Endometrial sampling via the hysteroscope is also feasible [7].

Cervical cancer incidence is increased in women with obesity versus those of normal weight [8]. The discrepancy may be partially due to lower cervical cancer screening rates in patients with obesity. Some have suggested that the lower screening rate in women with obesity is due to technical difficulties in obtaining the specimen [9, 10].

Little has been published on the effectiveness of different positons for pelvic examination in women with obesity. Visualization of the cervix was reportedly better in the super flexion position and permitted assessment for appropriateness for vaginal hysterectomy [11]. J. Marion Sims described use of the Sims speculum and Sims position in the mid 1800′s [12, 13]. Sims used the positon and the speculum to improve visualization of the upper vagina during vesicovaginal fistula repair. Originally, Sims position was described with the patient in an exaggerated left lateral position facing away from the examiner.

US gynecologic textbooks specify that the pelvic examination be performed in lithotomy position [14, 15]. Lateral decubitus (Sims) position is not mentioned. The training paradigm as reflected by the major authoritative sources for exam technique has likely led to adoption of lithotomy as the standard position for pelvic examination in the US. Lateral decubitus (Sims) position is used in the United Kingdom by a significant proportion of practitioners for routine pelvic examination [16]. Habit and geography seems to influence the choice of pelvic exam position rather than clinical circumstances.

The case series was limited by the small sample size. A larger sample might reveal more difficulties in cervical visualization or in patient acceptance. Furthermore, the case series represents the practice of one clinician, and may not be representative of results obtained more or less experienced gynecologists. The incidence of inability to visualize the cervix in dorsal lithotomy position was not measured for women with obesity in the author’s practice, limiting comparison with other clinicians’ experience.

## Conclusions

In a case series, the use of lateral decubitus position appears to improve visualization of the cervix in the outpatient setting among women with severe obesity without apparent adverse effect on staff or patient experience. Wider adoption of the technique may obviate the need for examination under anesthesia or other interventions in this patient population. As the obesity epidemic widens in scope, gynecologists will need to alter practice to meet the challenges presented by the anatomic alterations caused by the disease.

## Data Availability

The datasets during and/or analyzed during the current study are available from the corresponding author on reasonable request.

## Abbreviation

BMI: Body mass index
